# Association of sarcopenia with severe chemotherapy toxicities and survival in patients with advanced gastric cancer

**DOI:** 10.1093/oncolo/oyae123

**Published:** 2024-06-17

**Authors:** Wing-Lok Chan, Ho-Kwan Bryan Yun, Emina Edith Cheung, Michelle Liu, Li-Yu Hou, Ka-On Lam, Ian Yu-Hong Wong, Wan-Hang Keith Chiu, Simon Law, Dora Kwong

**Affiliations:** Department of Clinical Oncology, School of Clinical Medicine, LKS Faculty of Medicine, The University of Hong Kong, Pokfulam, Hong Kong; Department of Clinical Oncology, School of Clinical Medicine, LKS Faculty of Medicine, The University of Hong Kong, Pokfulam, Hong Kong; Department of Clinical Oncology, School of Clinical Medicine, LKS Faculty of Medicine, The University of Hong Kong, Pokfulam, Hong Kong; Department of Clinical Oncology, School of Clinical Medicine, LKS Faculty of Medicine, The University of Hong Kong, Pokfulam, Hong Kong; Department of Clinical Oncology, School of Clinical Medicine, LKS Faculty of Medicine, The University of Hong Kong, Pokfulam, Hong Kong; Department of Clinical Oncology, School of Clinical Medicine, LKS Faculty of Medicine, The University of Hong Kong, Pokfulam, Hong Kong; Department of Surgery, School of Clinical Medicine, LKS Faculty of Medicine, The University of Hong Kong, Pokfulam, Hong Kong; Department of Radiology, Queen Elizabeth Hospital, Jordan, Hong Kong; Department of Surgery, School of Clinical Medicine, LKS Faculty of Medicine, The University of Hong Kong, Pokfulam, Hong Kong; Department of Clinical Oncology, School of Clinical Medicine, LKS Faculty of Medicine, The University of Hong Kong, Pokfulam, Hong Kong

**Keywords:** chemotherapy toxicities, sarcopenia, gastric cancer, hematological toxicity, skeletal muscle mass

## Abstract

**Background:**

Sarcopenia or skeletal muscle depletion is a poor prognostic factor for gastric cancer (GC). However, existing cutoff values of skeletal muscle index (SMI) for defining sarcopenia have been found to have limitations when clinically applied. This study aimed to determine the optimal cutoff for SMI to predict severe toxicities of chemotherapy and overall survival (OS) in patients with advanced GC.

**Methods:**

Patients with metastatic gastric adenocarcinoma who received first-line palliative chemotherapy between January 2014 and December 2021 at Queen Mary Hospital, Hong Kong, were included in this study. The SMI was determined via a pre-chemotherapy computed tomography scan. Optimal cutoff points of SMI were identified by recursive partitioning analysis. Univariate and multivariate analyses evaluating risk factors of severe chemotherapy toxicities and OS were also performed.

**Results:**

A total of 158 patients (male: 108 (68.4%), median age: 65.3) were included. The SMI cutoff to define low SMI was ≤33 cm^2^/m^2^ for males and ≤28 cm^2^/m^2^ for females; 30 patients (19.0%) had low SMI. Patients with low SMI had a higher incidence of hematological toxicities (63.3% vs 32.0%, *P* = .001) and non-hematological toxicities (66.7% vs 36.7%, *P* = .003). Multivariable analysis indicated that low SMI and low serum albumin (≤28 g/L) were independent predictive factors of hematological toxicity, while low SMI and neutrophil-lymphocyte ratio ≥5 were predictive factors of non-hematological toxicity. Moreover, patients with low SMI had a significantly shorter OS (*P* = .011), lower response rate to chemotherapy (*P* = .045), and lower utilization of subsequent lines of treatment (*P* < .001).

**Conclusions:**

Using pre-chemotherapy SMI cutoff (≤33 cm^2^/m^2^ for males and 28 cm^2^/m^2^ for females) one can identify individuals with a higher risk of severe chemotherapy toxicities and worse prognosis.

## Teaser

Existing cutoff values of skeletal muscle index (SMI) for defining sarcopenia have limitations when clinically applied. This study aimed to determine the optimal cutoff for SMI to predict severe toxicities of chemotherapy and overall survival in patients with advanced gastric cancer.

**Implications for practice**

Using pre-chemotherapy SMI cutoff (SMI ≤ 33 cm^2^/m^2^ in males and ≤28 cm^2^/m^2^ in females) can identify individuals with a higher risk of severe chemotherapy toxicities and worse prognosis. Evaluation of skeletal muscle mass by CT imaging is a useful objective tool to identify individuals with low SMI. Physicians can consider using these cutoff points to identify patients who may have increased risk of chemotherapy toxicities and make decisions on dose adjustments, monitoring strategies after chemotherapy, and supportive interventions.

## Introduction

Gastric cancer (GC) is the world’s fourth leading cause of cancer mortality, particularly in East Asia.^1,2^ Despite the advancement in immunotherapy and targeted therapies, the prognosis of advanced GC remains poor, with median survival being approximately 10-14 months.^3^ Chemotherapy remains the backbone of systemic treatment in advanced GC. Patients with GC have a high prevalence of sarcopenia at the time of diagnosis, ranging from 12.5% to 57.7%.^4-7^ This high prevalence of sarcopenia is multifactorial—probably due to poor oral intake and malnutrition, use of chemotherapy with lack of physical activities, altered metabolism, and increased chronic inflammation within the body, thereby causing muscle wasting.^8,9^

Sarcopenia is a progressive and generalized loss of muscle mass and function and is associated with increased adverse outcomes, including falls, functional decline, disabilities, and mortality.^10^ Histologically, it is a condition that is prevalent in the older population, but it is now gaining attention as an important issue in the oncology field.^11-14^ Sarcopenia is highly prevalent in patients with cancer, with prevalence as high as 70%, and is strongly associated with worse overall survival (OS) and increased risk of adverse events, such as surgical complications and chemotherapy-related side effects.

While most previous studies focused on the associations of sarcopenia with surgical complications or chemotherapy toxicity in pre-operative settings, only a few studies have investigated the association between skeletal muscle mass and side effects of palliative chemotherapy or prognosis in patients with advanced GC. A Japanese study by Matsunaga et al revealed that patients with low skeletal muscle index (SMI) had a higher incidence of grade 3/4 side effects of chemotherapy and had a shorter survival rate than those with high SMI.^15^ However, the sample size was small (*n* = 83) and the chemotherapy regimens used were heterogeneous, with the involvement of triplet chemotherapies and intraperitoneal chemotherapy. Another Korean study by Lee et al revealed that patients with low SMI had a worse prognosis, but the study did not evaluate the side effects of chemotherapy.^16^ In addition, there is no universal cutoff point for low skeletal muscle mass, and most of the cutoff points used in extant literature have been derived based on Caucasian patients, thereby probably making these cutoff points inappropriate for Asian patients with cancer.^17-19^

The aim of our study was to examine the associations between skeletal muscle mass and chemotherapy toxicities and survival in patients with advanced or metastatic GC treated with first-line palliative chemotherapy and define a SMI cutoff point associated with the risk of severe toxicities and survival.

## Methods

### Patients

The medical records of consecutive patients diagnosed with advanced or metastatic GC between January 2014 and December 2021 in the Department of Clinical Oncology at Queen Mary Hospital, Hong Kong, were screened. The inclusion criteria were patients who had histologically proven adenocarcinoma of the stomach, with at least one metastatic lesion, received first-line palliative chemotherapy using fluoropyrimidine-based or platinum-based chemotherapy, had abdominal computed tomography (CT) or positron emission tomography-computed tomography (PET-CT) images within one month of beginning palliative chemotherapy, and accessible medical records in the Clinical Management System (CMS) under the Hospital Authority, Hong Kong. Patients with double primary cancers were excluded.

### Data collection

Clinical data were collected from the CMS under Hospital Authority, and included age, gender, Eastern Cooperative Oncology Group Performance Status (ECOG PS), height (m), body weight (kg), body mass index (BMI), comorbidities, histopathological type, site of primary tumor in the stomach, sites of metastasis, previous gastrectomy, previous radiotherapy, previous use of chemotherapy, type of chemotherapy and targeted agent, subsequent treatments, baseline laboratory results (including complete blood count, liver, and renal function test), albumin level, and any severe chemotherapy-related toxicity defined below, response to first-line chemotherapy according to RECIST 1.1 criteria, any use of second-line systemic treatment, and survival data.

### Chemotherapy-related toxicity

Severe chemotherapy-related toxicities included grade 3-5 hematological or non-hematological toxicities defined by the National Cancer Institute Common Toxicity Criteria for adverse events (NCI-CTCAE) V5.0. Patients were followed up for any severe chemotherapy-related toxicities for 3 weeks after completion of the first 6 cycles of chemotherapy or early termination of treatment or death. Data on termination of treatment due to toxicity was also collected.

### Assessment of skeletal muscle mass

Pre-chemotherapy CT images were used to assess skeletal muscle area (SMA). SMA (cm^2^) was quantified at the axial slice nearest the inferior aspect of the third lumbar vertebra (L3) by applying a Hounsfield units threshold range of −29 to +150. L3 was selected, as the cross-sectional area of the skeletal muscle in this region as it has been found to be most highly correlated with whole-body skeletal muscle mass.^20-22^ The muscles at the L3 level include the psoas major, erector spinae, quadratus psoas, transversus abdominis, external oblique abdominis, and internal oblique abdominis. The L3 skeletal muscle index (SMI, cm^2^/m^2^) was calculated as SMA normalized by the square of the height (m^2^). Skeletal muscle density (SMD) was expressed as the mean HU-value of the cross-sectional areas of the skeletal muscle. All images and calculations were analyzed using the MIM Maestro software version 7.0.

### Statistical analysis

In this study, continuous data are expressed as median with ranges, and categorical data are presented as counts and percentages. The Mann-Whitney *U* test was used to compare continuous variables. Categorical variables were compared using Fisher’s exact test or χ^2^ test.

The first step was to determine the association of SMI with the development of severe toxicities using an univariable logistic regression analysis. If the univariable analysis showed SMI was significantly associated with severe toxicities, the recursive partitioning analysis (RPA) was conducted to identify the optimal cutoff point of SMI to dichotomise patients into low SMI and normal SMI.^23^ Second, univariable and multivariable analysis were performed using logistic regression models to identify factors associated with severe hematological and non-hematological toxicities. The following clinical parameters were assessed: age, BMI, albumin level, neutrophil-lymphocyte ratio (NLR), any use of tube feeding, number of metastatic site, number of chemotherapy, any dose reduction of chemotherapy, number of comorbidities, low or normal SMI and SMD. Variables with *P* ≤ .1 in univariable analysis were added as confounders for multivariable analysis.

Further, OS was calculated from the first day of palliative chemotherapy to death by any cause. Patients who did not die were censored at the date of their last follow-up. The Kaplan-Meier method was used to estimate OS, and survival differences were examined using the log-rank test. Cox regression analysis was performed for significant findings regarding mortality. For all analyses, *P* < .05 was considered statistically significant. Data analysis was performed using R software (version 3.3.0+).

### Ethics approval

This retrospective study was approved by the Institutional Review Board of The University of Hong Kong/Hospital Authority Hong Kong West Cluster (HKU/HA HLW IRB; ref no. UW 20-345).

## Results

### Characteristics of the study population

This study included 158 consecutive patients (Table 1), among which 108 (68.4%) were male and 121 (76.6%) had de-novo metastasis. The median age was 65.3 years (range 26.3-88.4 years). The average BMI was 21.4 kg/m^2^ and 36 patients (38.1%) were underweight (BMI < 18.5 kg/m^2^). All patients had an ECOG status of 0 or 1. In addition, 21 patients (13.3%) were HER2-positive.

**Table 1.** Patients characteristics.

	Patients (total: 158)	Percentage
Age (years)		
Median (range)	65.3 (26.3-88.4)	
ECOG		
0	10	8.9
1	148	91.1
Male	108	68.4
BMI (kg/m^2^)		
Average (range)	21.4 (14.3-36.9)	22.8
< 18.5 kg/m^2^	36	
Recurrence		
Recurrent	37	23.4
De novo	121	76.6
Previous gastrectomy	50	31.6
Previous palliative radiotherapy to stomach	6	3.8
HER2 positive	21	13.3
Primary tumor location		
Cardia	53	33.5
Fundus	12	7.6
Body	75	47.5
Antrum	50	31.6
Pylorus	24	15.2
Linitis plastic	13	8.2
Site of metastasis		
Liver	37	23.4
Peritoneum	103	65.2
Distant lymph nodes	94	59.4
Lung	22	13.9
Bone	17	10.8
Adrenal	8	50.6
Brain	1	0.6
Number of metastatic sites		
1	69	43.0
2	59	37.3
3	25	15.8
4	6	3.8
Need tube feeding	11	7.0
Comorbidities		
Diabetes	34	21.5
Hypertension	63	39.9
Hyperlipidemia	36	22.8
Ischemic heart disease	17	10.8
Cerebrovascular disease	4	2.5
Hepatitis B carrier	12	7.6
No. of comorbidities		
0	69	43.7
1	35	22.2
2	27	17.1
3	20	12.7
4	7	4.4
NoNo. of chemotherapy		
Mono-chemotherapy	15	9.5
Doublet chemotherapies	143	90.5
Dose reduction at first cycle	44	27.8

Further, all patients received 5-FU-based chemotherapy; 15 (9.5%) underwent monotherapy using TS-ONE, while 143 (90.5%) underwent doublet chemotherapy (Supplementary Table S1). In addition, 21 (13.3%) had HER2-positive tumors and received trastuzumab along with chemotherapy. The median number of cycles of chemotherapy received was 5 (range 1-28). Further, 44 (27.8%) patients underwent dose reduction at the initial cycle, while 114 (72.2%) received the standard dose.

### Skeletal muscle measurement

The median SMI was 37.2 cm^2^/m^2^ (range 14 cm^2^/m^2^-72.7 cm^2^/m^2^) for all patients, 39.9 cm^2^/m^2^ for male patients (range 14 cm^2^/m^2^-72.7 cm^2^/m^2^), and 34.1 cm^2^/m^2^ (range 19.9 cm^2^/m^2^-50.4 cm^2^/m^2^) for female patients. On univariable analysis, SMI was associated with both severe hematological (HR 0.83, 95% CI, 0.75-0.96, *P* = .03) and non-hematological toxicities (HR 0.97, 95% CI, 0.85-0.99, *P* = .04).

After RPA, low SMI was defined with the cutoff points of 33 cm^2^/m^2^ for males and 28 cm^2^/m^2^ for females. Further, 30 patients (19.9%) were identified as having low SMI and 128 (81%) as having normal SMI. The mean pre-treatment SMD for the entire group was 37.8 HU (range: −2.72 HU to 66.24 HU).

### Types and frequency of severe chemotherapy toxicities

Overall, 95 out of 158 patients (60.1%) developed severe toxicities after chemotherapy (Table 2). Sixty percent (38.0%) patients had G3-5 hematological toxicities and 67 (42.4%) had G3-5 non-hematological toxicities. The most common hematological toxicity was anemia (*n* = 36; 22.8%), while the most common non-hematological toxicities were hyponatremia (*n* = 21; 16.4%) and infection (*n* = 21; 16.4%).

**Table 2.** The incidence of grades 3-5 toxicities in patients with low SMI and normal SMI.

	All patients (*n* = 158)	Low SMI (*n* = 30)	Normal SMI (*n* = 128)	*P*-value
Hematological AE	**60 (38%)**	**19 (63.3%)**	**41 (32.0%)**	***.001***
Anemia	36 (22.8%)	16 (53.3%)	20 (15.6%)	**<.001**
Neutropenia	33 (20.9%)	10 (33.3%)	23 (18%)	.083
Thrombocytopenia	12 (7.6%)	6 (20.0%)	6 (4.7%)	**.004**
Neutropenic fever	12 (7.6%)	5 (16.7%)	7 (5.5%)	**.037**
Non-hematological AE	**67 (42.4%)**	**20 (66.7%)**	**47 (36.7%)**	**.003**
Hyponatremia	31 (19.6%)	10 (33.3%)	21 (16.4%)	**.044**
Hypokalemia	20 (12.7%)	7 (23.3%)	13 (10.2%)	.067
Hyperkalemia	2 (1.3%)	2 (6.7%)	0 (0%)	**.035**
Hypercalcemia	1 (0.6%)	1 (3.3%)	0 (0%)	.190
Creatinine increased	10 (6.3%)	3 (10.0%)	7 (5.5%)	.402
ALT increased	3 (1.9%)	0 (0%)	3 (2.3%)	.662
AST increased	4 (2.5%)	1 (3.3%)	3 (2.3%)	1.120
Infection	21 (13.3%)	9 (30.0%)	12 (9.4%)	**.006**
Hand-foot syndrome	7 (4.4%)	4 (13.3%)	3 (2.3%)	**.009**
Nausea/vomiting	13 (8.2%)	4 (13.3%)	9 (7.0%)	.258
Diarrhea	15 (9.5%)	5 (16.7%)	10 (7.8%)	.136

Bold values indicates significant differences.

### Association of SMI with severe toxicities

#### Differences in the incidences of severe toxicities in low SMI vs normal SMI

The overall grade 3-5 hematological toxicities occurred significantly more often in patients with low SMI than patients with normal SMI (63.3% vs 32.0%, *P* = .001). The incidences of anemia (53.3% vs 15.6%, *P* < .001), thrombocytopenia (20.0% vs 4.7%, *P* = .004), and neutropenic fever (16.7% vs 5.5%, *P* = .037) were all significantly higher in patients with low SMI. In addition, for grade 3-5 non-hematological toxicities, the incidence was significantly higher in patients with low SMI than in patients with normal SMI (66.7% vs 36.7%, *P* = .003). Hyponatremia, infection, hyperkalemia, and hand-foot syndrome were all more prevalent in patients with low SMI. The incidences of other side effects—including deranged renal function, liver function, diarrhea, and nausea/vomiting—were not significantly different between the 2 groups.

#### Univariable and multivariable analyses on severe toxicities

The results of the univariate and multivariate analyses are presented in Table 3. After univariable and multivariable analyses, low SMI and albumin ≤ 28 g/L were identified as independent predictors of severe hematological toxicities, while low SMI and neutrophil-lymphocyte ratio (NLR) ≥ 5 were identified as independent predictors of severe non-hematological toxicities.

**Table 3.** Univariable and multivariable analysis on the variables that were associated with severe hematological and non-hematological toxicities.

	Severe hematological toxicities						Severe non-hematological toxicities					
	Univariable analysis			Multivariable analysis			Univariable analysis			Multivariable analysis		
	Odd ratio	95% CI	*P*-value	Odd ratio	95% CI	*P*-value	Odd ratio	95% CI	*P*-value	Odd ratio	95% CI	*P*-value
Age (≥ 70)	1.21	0.96-1.79	.611				1.15	0.86-1.89	.774			
Low-SMI	3.67	1.60-8.41	**.002**	2.81	1.17-6.73	**.021**	3.45	1.49-7.98	**.004**	2.53	1.01-6.30	**.047**
SMD (HU)	1.03	0.99-1.06	.191				1.02	0.98-1.06	.328			
Albumin (≤ 28g/L)	4.15	1.22-14.13	**.023**	2.77	0.76-10.17	.125	3.38	0.99-11.48	**.051**	1.39	0.34-5.72	.647
BMI (≤ 18kg/m^2^)	1.83	0.80-4.16	.152				1.45	0.64-3.30	.372			
NLR (≥ 5)	1.51	0.74-3.10	.259				3.95	1.89-8.35	**<.001**	3.13	1.40-6.98	**.005**
Doublets chemotherapy	0.86	0.31-2.40	.774				0.62	0.25-1.71	.355			
Number of comorbidities (0 as ref.)												
1	0.81	0.35-1.90	.63				1.16	0.51-2.62	.725			
2	0.78	0.31-1.98	.598				0.84	0.34-2.08	.710			
3	1.27	0.47-3.48	.638				0.41	0.13-1.25	.117			
4	1.17	0.24-5.63	.848				0.92	0.19-4.42	.916			
Number of metastatic site (1 site as ref.)												
2	1.18	0.58-2.41	.651				0.98	0.48-1.99	.955			
3	0.97	0.37-2.51	.946				1.32	0.53-3.31	.556			
4	0.86	0.15-5.04	.867				1.43	0.27-7.60	.676			
Dose reduction	1.04	0.51-2.13	.915				0.92	0.45-1.86	.813			
Use of tube feeding	4.87	1.24-19.16	**.023**	3.66	0.88-15.22	.074	6.91	1.44-33.11	**.016**	4.46	0.86-23.18	.075

### Duration of treatment, treatment response, and overall survival

Patients with low SMI received fewer cycles of chemotherapy compared with patients with normal SMI (median number of cycles: 3 vs 6, *P* < .001). More patients with low SMI underwent early termination of treatment (≤ 3 cycles of chemotherapy) than patients with normal SMI (76.6% vs 43.0%, *P* = .001). The overall response rate (10.0% vs 27.3%, *P* = .045) and use of subsequent lines of treatment (26.7% vs 60.9%, *P* < .001) were significantly lower in patients with low SMI than patients with normal SMI.

**Figure 1.**
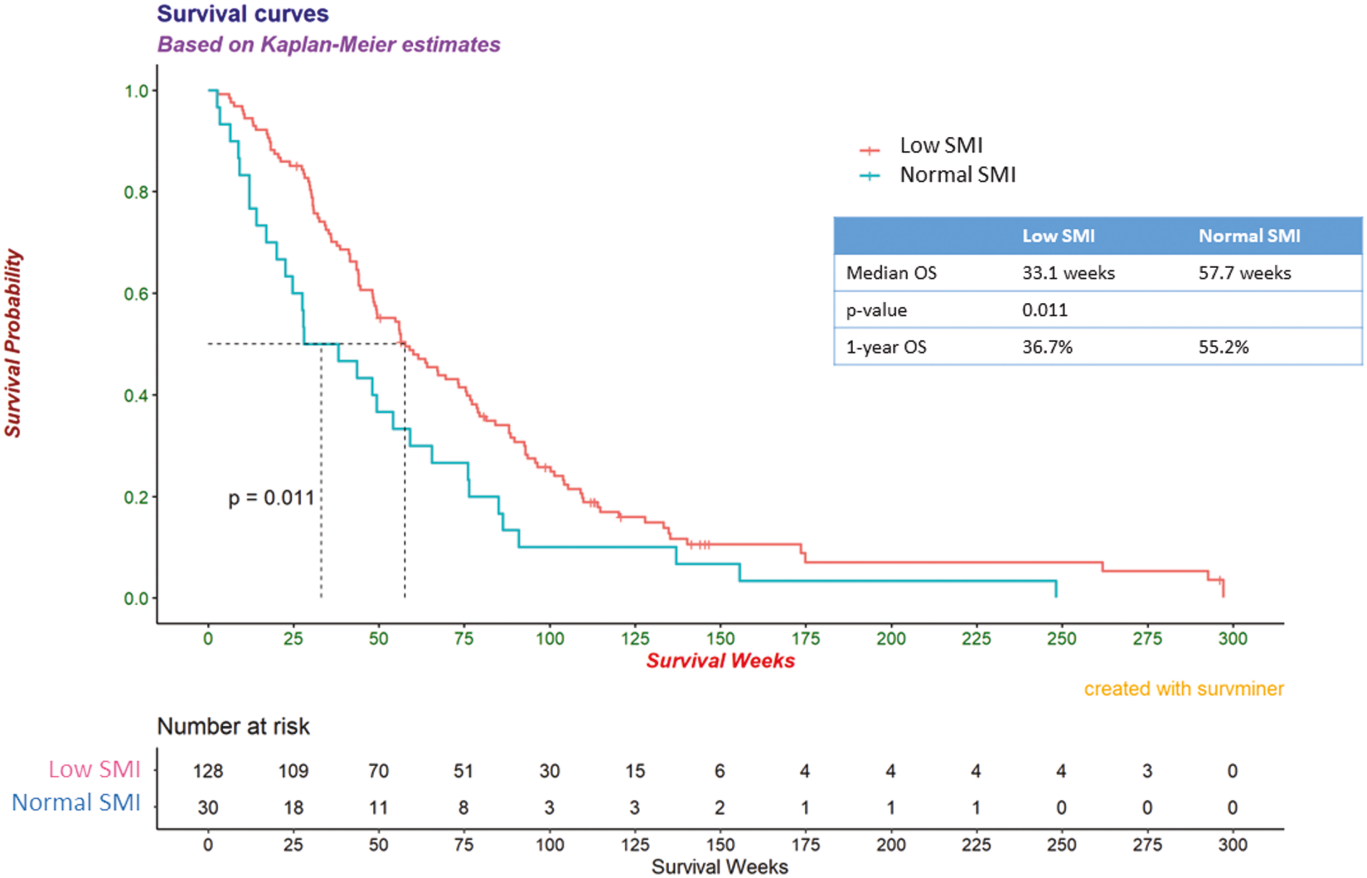
Kaplan-Meier curves for survival time in patients with low SMI and normal SMI.

The median follow-up period for all patients was 40 months. Patients with low SMI had a significantly shorter OS than those with normal SMI (33.1 vs 57.7 weeks, *P* = .011) (Figure 1). Further, univariable and multivariable analysis revealed that low SMI (HR 1.68, 95% CI, 1.08-2.61, *P* = .02), albumin ≤ 28 g/L (HR 2.18, 95% CI, 1.18-4.01, *P* = .01), and NLR ≥ 5 (HR 1.98, 95% CI, 1.32-2.95, *P* < .001) were significantly associated with shorter OS (Supplementary Table S2).

## Discussion

Currently, there are different definitions of sarcopenia and a wide variability exists in the cutoff values of SMI for defining low muscle mass. Instead of using the existing cutoff points, we decided to identify the cutoff points that can potentially guide our GC management plan. There are several reasons for this decision.

First, the commonly used definitions were developed for non-Asians and might be inappropriate for the Asians. The SMI is a measure of muscle mass relative to body height, weight, or BMI. The 3 most commonly used definitions in extant studies—those given by the European Working Group on Sarcopenia in Older People (EWGSOP2), Martin et al, and Prado et al—were developed on the basis of healthy European adults and cancer patients in Canada.^10,17,18,24^ The Europeans and the Canadians have a bigger body build and larger skeletal dimensions than Asians. When applying these cutoff points, previous studies have revealed that the prevalence of sarcopenia or low SMI was significantly higher in Asian than Caucasians, thereby suggesting that these cutoff values may not be accurate for Asian populations.^25-27^

Second, the optimal SMI cutoff points may vary with different cancer types and stages. The meta-analysis by Au et al found that the association between low lean mass and cancer mortality was insignificant in certain types of cancer—for example, breast cancer, prostate cancer, and ovarian cancer.^28^ Moreover, the studies included in their meta-analysis also used different cutoff points. Therefore, it is important to identify specific cutoff values for each cancer type instead of using a single universal cutoff point.

Third, the existing cutoff points of SMI for GC developed in other studies were based on either the standard deviations of the study populations or the cancer survival rate of that cohort. For example, in Zhuang et al’s study, SMI cutoff points of 34.9 cm^2^/m^2^ for females and 40.8 cm^2^/m^2^ for males were found based on the survival differences with log-rank test in 937 patients with stages I-III GC and undergoing gastrectomy.^7^ In Sakurai et al’s study, which included 569 patients who underwent gastrectomy, the cutoff of sarcopenia (43.2 cm^2^/m^2^ for males and 34.6 cm^2^/m^2^ for females) was determined by the first quartile of the distribution in the study population.^29^ In Matsunaga et al’s study, which investigated 83 patients receiving first-line 5-FU chemotherapy, the SMI cutoff of 45.1 cm^2^/m^2^ for males and 34.5 cm^2^/m^2^ for females was developed by dividing the cohort into half; 41 patients had low SMI and 42 patients had high SMI. Although studies confirmed the significant associations of these cutoff points with survival in patients with GC, it is unclear how these cutoff points can guide physicians regarding subsequent treatment plans.

In our study, the SMI cutoff points of <33 cm^2^/m^2^ for males and <28 cm^2^/m^2^ for females predict severe chemotherapy toxicities and are associated with worse survival outcomes in patients with advanced GC on palliative chemotherapy. Patients with low SMI had a significantly higher risk of grade 3/4 hematological and non-hematological toxicities. Moreover, these patients had a significantly shorter survival rate, lower response rate to first-line chemotherapy, and less frequent use of subsequent lines of treatment. The cutoff point of SMI found in our study was determined by the occurrence of severe toxicities and mortality; thus, it has both prognostic and predictive significance in a clinical context. For patients with SMI below our cutoff point, the risks of severe hematological toxicities and non-hematological toxicities were doubled. Over half of the patients with sarcopenia experienced early termination of treatment. Further, the response to first-line systemic treatment was rather low, only approximately 10%, and the median survival was only approximately 33.1 weeks. According to histological data, the median survival duration of patients with advanced GC who did not receive systemic treatment was approximately 4.3 months (ie, 30.1 weeks) and this duration is similar to the median OS of patients with low SMI.

Therefore, given the much higher risk of severe toxicities and minimal benefit in terms of response and survival rates in patients with sarcopenia, it is important for both patients and caregivers to understand the pros and cons of treatment as well as the poor prognosis before beginning chemotherapy. An honest and forthright communication among physicians, patients, and caregivers is essential to understand the goal of treatment and avoid false hope and deterioration of quality of life for the patient. Moreover, our study also revealed that low serum albumin level was associated with higher risk of severe hematological toxicities and shorter OS. Sarcopenia and low serum albumin level synergistically increase the risk of incident disability in older adults. Early identification of people at risk for malnutrition and early intervention is important in patients with advanced GC. Adequate nutritional support with a high protein diet and addition of multivitamins and omega-3 fatty acids in combination with close monitoring of acute severe toxicities may improve a patient’s tolerance to chemotherapy. Future prospective research should focus on intervention studies to evaluate the effectiveness of nutritional interventions and benefits of nutritional supplements in improving the outcomes of patients with sarcopenia.

Our study has several limitations. First, this is a retrospective study with patients only from a single institution. All the data were collected from the patients’ records. Second, since sarcopenia involved 3 aspects—muscle strength, muscle quantity/ quality, and physical performance, the data on muscle strength and physical performance (eg, grip strength, gait speed, or balancing ability) were not collected. Third, data on inflammatory markers (eg, C-reactive protein (CRP), interleukin-6 (IL-6)) were not available while these markers might correlate with the body inflammatory status and sarcopenia. Fourth, data on low-grade toxicities were not collected in this study. These low-grade toxicities also likely affect the health-related quality of life in advanced cancer patients. Fifth, we did not collect data on the longitudinal changes in the SMI during the course of chemotherapy. The change in SMI during chemotherapy may also affect chemotherapy tolerance and survival of patients.

Nevertheless, our study also had several strengths. First, to the best of our knowledge, this is one of the largest retrospective studies that involves only patients with advanced or metastatic GC on palliative chemotherapy. Second, the included patients were rather homogenous, as they all had pre-treatment CT images in DICOM format and had received first-line palliative chemotherapy using platinum-based chemotherapy or 5-FU based chemotherapy. Third, we developed a comprehensive collection of data from the patients’ medical records; patients with missing data were excluded from the analysis.

## Conclusion

We observed that low SMI (SMI ≤ 33 cm^2^/m^2^ in males and ≤ 28 cm^2^/m^2^ in females) is an independent predictive factor for severe toxicities in patients with advanced GC undergoing chemotherapy and is an important prognostic factor for survival. Evaluation of skeletal muscle mass by CT imaging is a useful objective tool to identify individuals with low SMI. Physicians can consider using these cutoff points to identify patients who may have increased risk of chemotherapy toxicities and make decisions on dose adjustments, monitoring strategies after chemotherapy, and supportive interventions. Further prospective research is warranted to evaluate the effectiveness of nutritional therapies in reversing the condition of sarcopenia and improving the prognosis of patients with advanced GC.

## Supplementary material

Supplementary material is available at *The Oncologist* online.

oyae123_suppl_Supplementary_Tables

## Data Availability

The data underlying this article will be shared on reasonable request to the corresponding author.
