# Supplementary material for: Association of sarcopenia with severe chemotherapy toxicities and survival in patients with advanced gastric cancer
Source: Oncologist. 2024 Jun 17;29(10):e1272–9. doi: 10.1093/oncolo/oyae123 (PMC11449055; doi:10.1093/oncolo/oyae123)
Supplement: oyae123_suppl_Supplementary_Tables [file oyae123_suppl_supplementary_tables.docx]

**Supplementary Table 1: chemotherapy regimens used in the low SMI and normal SMI groups**

|  | **All Patients**  **(n = 158)** | **Low SMI**  **(n = 30)** | **Normal SMI**  **(n = 128)** | ***P-value*** |
| --- | --- | --- | --- | --- |
| **Monotherapy** | 15 (9.5%) | 3 (10.0%) | 12 (9.4%) | *0.916* |
| - TS-1 | 15 (9.5%) | 3 (10.0%) | 12 (9.4%) |  |
| **Combination chemotherapy** | 143 (90.5%) | 27 (90.0%) | 116 (90.6%) |  |
| - Carboplatin & 5-FU (CF) | 2 (1.3%) | 1 (3.3%) | 1 (0.8%) |  |
| - Carboplatin & TS-1 | 1 (0.6%) | 0 (0%) | 1 (0.8%) |  |
| - Carboplatin & Xeloda (CX) | 13 (8.2%) | 2 (6.7%) | 11 (8.6%) |  |
| - Cisplatin & 5-FU (PF) | 3 (1.9%) | 0 (0%) | 3 (2.3%) |  |
| - Cisplatin & TS-1 (SP) | 10 (6.3%) | 2 (6.7%) | 8 (6.3%) |  |
| - Cisplatin & Xeloda (PX) | 54 (34.2%) | 10 (33.3%) | 44 (34.4%) |  |
| - Oxaliplatin & TS-1 (SOX) | 3 (1.9%) | 0 (0%) | 3 (2.3%) |  |
| - CapOx | 39 (24.7%) | 6 (20.0%) | 33 (25.8%) |  |
| - FOLFOX | 3 (1.9%) | 3 (10.0%) | 0 (0%) |  |
| - FLOT | 15 (9.5%) | 3 (10.0%) | 12 (9.4%) |  |
| **Dose at first cycle** |  |  |  | *0.77* |
| - Dose Reduction | 44 (27.8%) | 9 (30%) | 35 (27.3%) |  |
| - Standard dose | 114 (72.2%) | 21 (70%) | 93 (72.7%) |  |

**Supplementary Table 2: Univariable and multivariable analysis on factors associated with overall survival**

|  | **Univariable Analysis** | | | **Multivariable Analysis** | | |
| --- | --- | --- | --- | --- | --- | --- |
|  | **Hazard ratio** | **95% CI** | **P-value** | **Hazard ratio** | **95% CI** | **P-value** |
| Age (≥70) | 1.17 | 0.92 – 1.19 | 0.26 |  |  |  |
| Low-SMI | 1.68 | 1.12 – 2.52 | **0.012** | 1.68 | 1.08 – 2.61 | **0.02** |
| SMD (HU) | 1.00 | 0.96 – 1.04 | 0.975 |  |  |  |
| Albumin (≤ 28 g/L) | 2.98 | 1.66 – 5.34 | **<0.001** | 2.18 | 1.18 – 4.01 | **0.012** |
| BMI (≤ 18 kg/m^2^) | 1.12 | 0.76 – 1.66 | 0.570 | 0.98 | 0.64 – 1.50 | 0.931 |
| NLR (≥ 5) | 1.96 | 1.34 – 2.87 | **<0.001** | 1.98 | 1.32 – 2.95 | **<0.001** |
| Doublets chemotherapy | 0.99 | 0.56 – 1.77 | 0.979 |  |  |  |
| Number of comorbidities  (0 as ref.) |  |  |  |  |  |  |
| - 1 | 1.13 | 0.74 – 1.72 | 0.578 |  |  |  |
| - 2 | 1.01 | 0.64 – 1.60 | 0.971 |  |  |  |
| - 3 | 0.81 | 0.47 – 1.40 | 0.448 |  |  |  |
| - 4 | 0.61 | 0.25 – 1.539 | 0.298 |  |  |  |
| Number of metastatic sites  (1 site as ref.) |  |  |  |  |  |  |
| - 2 | 1.10 | 0.76 – 1.58 | 0.624 |  |  |  |
| - 3 | 1.11 | 0.67 – 1.82 | 0.691 |  |  |  |
| - 4 | 1.62 | 0.70 – 3.76 | 0.261 |  |  |  |
| Dose reduction | 1.57 | 1.09 – 2.25 | **0.015** | 1.24 | 0.86 – 2.21 | 0.352 |
| Use of tube feeding | 1.34 | 0.70 – 2.58 | 0.375 |  |  |  |
